# Post-traumatic endophthalmitis caused by streptococcus species in preschool children: clinical features, antibiotic susceptibilities and outcomes

**DOI:** 10.1038/s41433-021-01449-6

**Published:** 2021-02-24

**Authors:** Yao Yang, Wenjun Sui, Fang Duan, Yujie Li, Jieting Zeng, Zhaoxin Jiang, Manli Liu, Zhaohui Yuan, Xiaofeng Lin

**Affiliations:** State Key Laboratory of Ophthalmology, Zhongshan Ophthalmic Center, Sun Yat-sen University, Guangzhou, China

**Keywords:** Eye diseases, Trauma, Infectious diseases

## Abstract

**Background/Objectives:**

Streptococcus is a common cause of post-traumatic endophthalmitis in children. This study aimed to analyse the clinical features, antibiotic susceptibilities and outcomes of traumatic endophthalmitis caused by streptococcus in preschool children.

**Subjects/Methods:**

Patients aged ≤6 years with traumatic streptococcal endophthalmitis seen at Zhongshan Ophthalmic Center between January 2013 and December 2018 were included in this retrospective study.

**Results:**

In total, 21 patients (21 eyes) were included. The mean age of the patients was 3.3 ± 1.7 years, where 57.1% were males. Scissors (28.6%, *n* = 6) were the most common cause of injury; 86.7% of patients were injured at home. Zone I (80.9%) was the most common wound site; 90.5% of patients presented with a traumatic cataract. In general, *Streptococcus pneumoniae* (47.6%) was the most common isolate. Viridans group streptococci accounted for 58.3% of cases in children aged 0–3 years, while *S. pneumoniae* accounted for 66.7% of cases in children aged 4–6 years. The susceptibility rates of streptococcus to cefuroxime, levofloxacin and ofloxacin were 100%, 95.0% and 90.5%, respectively. Although all the patients underwent vitrectomy combined with silicone oil tamponade, the final visual outcomes were no better than counting fingers.

**Conclusions:**

Although *S. pneumoniae* was the most prevalent organism in general, the dominant species varied between different age groups. The commonly used antibiotics, cefuroxime and fluoroquinolone, showed higher antibiotic susceptibility. Despite prompt treatment, the visual outcomes of paediatric post-traumatic endophthalmitis in preschool children were poor.

## Introduction

Endophthalmitis is a rare but sight-threatening complication in patients with eye trauma [1, 2]. Its incidence rate is estimated to be 2.8–54.2% in children with ocular trauma, varying geographically [1, 3, 4].

The *Streptococcus* species is a common pathogen causing endophthalmitis. In adult-dominated populations, the streptococcus species is the leading cause of endophthalmitis after glaucoma filtering surgery. This species is also the common genus of organisms identified in endophthalmitis post cataract surgery and post intravitreal injection [5–8]. Among patients with streptococcal endophthalmitis, *Streptococcus pneumoniae*, viridans group streptococcus and group B β-haemolytic streptococcus were the main isolates, although the dominant isolates varied in different studies [8–11]. Previous studies have focused on paediatric post-traumatic endophthalmitis caused by all pathogens [12–14]. They found that the streptococcus species was a common cause of post-traumatic endophthalmitis in children. Thordsen et al. [2] reported that 29% of isolates were streptococcus in their study on paediatric post-traumatic endophthalmitis, and in another study, Al-Rashaed and Abu El-Asrar [15] found that streptococcus accounted for 53.7% of cases. Studies on adult-dominated streptococcal endophthalmitis found that despite prompt treatment, the visual outcomes were mostly poor, and about 24.7–33.3% of cases resulted in evisceration or enucleation [9, 11]. However, there is insufficient information on streptococcal endophthalmitis in the paediatric population, hence it is necessary to conduct research on paediatric post-traumatic endophthalmitis caused by streptococcus.

This study retrospectively analysed the clinical characteristics, antibiotic susceptibilities and visual outcomes of post-traumatic streptococcal endophthalmitis in affected preschool children, providing valuable information on the clinical management of the disease.

## Subjects and methods

### Population

Consecutive medical records of all patients ≤6 years old with culture-proven streptococcal traumatic endophthalmitis admitted to Zhongshan Ophthalmic Center, Guangzhou, in southern China, between January 2013 and December 2018, were retrospectively reviewed. This study was performed in compliance with the principles of the Declaration of Helsinki and was approved by the Institutional Ethics Committee of Zhongshan Ophthalmic Center, Sun Yat-sen University. The requirement for patient consent was waived, given the retrospective nature of the study.

### Procedures

Personal information, including age and sex, the location where the injury occurred, the mechanism of injury and the duration from injury-to-primary repair were recorded. Each patient underwent ophthalmic examination conducted by an ophthalmologist. The characteristics of injury and endophthalmitis were obtained from the electronic medical record system. Data on medical treatment consisting of topical and intravenous use of antibiotics were recorded. Penicillin, cephalosporin, clindamycin and vancomycin were chosen for intravenous injection at the discretion of the ophthalmologist. Clindamycin was used when patients were allergic to cephalosporins. Surgical treatment, including intravitreal injection of vancomycin and pars plana vitrectomy combined with silicone oil tamponade, was performed based on the ophthalmologist’s assessment. The average hospitalisation time and the average time from admission to vitrectomy were recorded. The visual acuity and silicone oil removal/non-removal were recorded at the last follow-up.

An aqueous/vitreous tap for culture was performed on the patients clinically diagnosed with endophthalmitis during surgery. The aqueous humour from the anterior chamber was aspirated through the corneal limbus with a needle attached to a 1-ml syringe. Vitreous specimens were collected through the pars plana prior to antibiotic injection or vitrectomy using a needle or vitrector. Samples were inoculated into bacterial and fungal media [16]. All bacterial isolates were subjected to species identification on the Vitek 2 Compact (BioMerieux, Inc., Marcy-l′Étoile, France). Antibiotic susceptibility testing of the isolated bacteria was performed using either minimal inhibitory concentration (MIC) methods or the Kirby–Bauer disc diffusion method, depending on the antibiotics. The antibiotic susceptibility was determined using the methods of the Clinical and Laboratory Standards Institute. The following antibiotics were tested in the susceptibility analysis: fluoroquinolones (levofloxacin and ofloxacin), cephalosporins (ceftazidime, cefazolin, cefoxitin and cefuroxime), aminoglycosides (tobramycin and neomycin), penicillin, azithromycin and chloramphenicol.

### Definitions

The population was divided into two groups based on patients’ age: 0–3 years and 4–6 years. The locations where the injury occurred were classified as either at home or in public, and the injury mode was classified as either self-injury or other-inflicted injury. The wound sites were categorised as zone I (wound limited to the corneal area, including the corneoscleral limbus), zone II (5 mm posterior to the corneoscleral limbus) and zone III (posterior to the anterior 5 mm of the cornea) [17]. Streptococci were further subdivided into *S. pneumoniae*, viridans group streptococci and *S. pluranimalium*. The cases were classified into concurrent endophthalmitis, which was defined as endophthalmitis diagnosed at presentation concurrently with an open globe, and post-open globe repair endophthalmitis, which was defined as eyes diagnosed with endophthalmitis after primary repair [18].

### Statistical analysis

Statistical analysis was performed using SPSS version 16.0 (SPSS, Chicago, IL, USA). Characteristics of the study population and the isolates and the susceptibility rate were summarised using means and standard deviations for continuous variables and percentages for categorical variables. Differences in categorical variables were assessed using the Fisher exact test. Results with *P* < 0.05 were considered statistically significant.

## Results

Twenty-one preschool children (≤6 years old) (21 eyes) with streptococcal endophthalmitis after ocular trauma were enrolled in this study. The mean age of the patients was 3.3 ± 1.7 years (range: 8 months–6 years) and included 11 males (57.1%). The demographic information, causes of injury, treatment measures and visual outcomes of participants are listed in Table 1.

**Table 1:** Management and visual outcome of paediatric streptococcal endophthalmitis by case.

Case	Age	Sex	Causes of trauma	Days from injury to primary repair	Concurrent endophthalmitis	Presenting visual acuity	Intravenous antibiotics	Intravitreal injection	Vitrectomy + silicone oil	Final VA
1	8 m	M	Plant branch	1	Yes	**-**	Yes	No	Yes	**-**
2	1 y	F	Metal wire	3	Yes	**-**	Yes	No	Yes	**-**
3	1 y	F	Scissors	0	Yes	LP	Yes	No	Yes	**LP**
4	1 y	F	Scissors	-	No	**-**	Yes	No	Yes	**-**
5	2 y	M	Uncertain	2	Yes	**-**	Yes	No	Yes	**-**
6	2 y	M	Plant branch	2	Yes	**-**	Yes	No	Yes	**-**
7	3 y	M	Metal nail	8	Yes	LP	Yes	Yes	Yes	**-**
8	3 y	M	Scissors	0	No	**-**	Yes	No	Yes	**-**
9	3 y	M	Toy	0	No	**-**	Yes	No	Yes	**-**
10	3 y	M	Bird	4	Yes	HM	Yes	Yes	Yes	LP
11	3 y	M	Scissors	5	Yes	**-**	Yes	Yes	Yes	**-**
12	3 y	F	Plant branch	-	Yes	**-**	Yes	No	Yes	**-**
13	4 y	F	Knife	Self sealed	Self sealed	CF	Yes	No	Yes	HM
14	4 y	M	Metal wire	Self sealed	Self sealed	**-**	Yes	No	Yes	LP
15	5 y	F	Toy	Self sealed	Self sealed	CF	Yes	Yes	Yes	CF
16	5 y	F	Scissor	1	Yes	**-**	Yes	Yes	Yes	HM
17	5 y	M	Metal stick	0	No	NLP	Yes	No	Yes	NLP
18	5 y	M	Scissors	4	Yes	NLP	Yes	Yes	Yes	HM
19	6 y	F	Metal nail	6	Yes	NLP	Yes	Yes	Yes	-
20	6 y	M	Toy	2	No	**-**	Yes	No	Yes	LP
21	6 y	F	Injection syringe	Self sealed	Self sealed	**-**	Yes	No	Yes	HM

Scissors (28.6%, *n* = 6) were the most common cause of injury, followed by toys and plant branches (both 14.3%, *n* = 3). Only one patient (4.8%) presented with an intraocular foreign body. Besides the two patients whose records lacked primary repair information because of referral, four patients (21.1%) presented with a self-sealed wound after injury and 15 patients (78.9%) underwent wound repair. The average duration of injury-to-primary repair was 2.6 ± 2.4 days. Among the patients, 57.1% were diagnosed with endophthalmitis concurrent with primary repair, and 23.8% of the patients developed endophthalmitis after primary repair. The average length of hospitalisation was 7.6 ± 4.1 days. All patients received intravenous antibiotics: five received cephalosporin, three received cephalosporin combined with vancomycin, 11 received cephalosporin combined with clindamycin, one patient was treated with penicillin and one patient received clindamycin because of an allergy to cephalosporins. All patients underwent pars plana vitrectomy with silicone oil tamponade, and the average time from admission to vitrectomy was 1.8 ± 2.5 days. Intravitreal vancomycin injection prior to vitrectomy was administered in seven patients (33.3%). None of these patients developed panophthalmitis or orbital cellulitis, and none developed evisceration or enucleation due to uncontrollable infection.

The clinical characteristics are summarised in Table 2. Eighteen children (85.7%) injured themselves and three (14.3%) were injured by others. The medical records of 15 patients recorded the location of the injury, among whom 86.7% were injured at home. Zone I was the most common wound site (80.9%), vitreous opacification (95.2%), traumatic cataract (90.5%) and hypopyon (71.4%) were the top three common clinical symptoms. Retinal detachment occurred in 23.8% of patients.

**Table 2:** Injury aninformation d clinical characteristics of paediatric streptococcal endophthalmitis.

Variables	Findings
Injury characteristics	
Location of injury*	
Home	13/15 (86.7%)
Public places	2/15 (13.3%)
Injury condition	
Injured by others	3/21 (14.3%)
Injured themselves	18/21 (85.7%)
Mean duration of injury-to-primary repair	2.6 ± 2.4 day (*n* = 16*, range: 0–8)
Type of injury	
Penetrating	20/21 (95.2%)
Intraocular foreign body injury	1/21 (4.8%)
Time of diagnosis	
Concurrent endophthalmitis	12/21 (57.1%)
Post-OGR endophthalmitis	5/21 (23.8%)
Self-sealed	4/21 (19.1%)
Clinical characteristics	
Location of wound	
Zone I	17/21 (80.9%)
Zone II	3/21 (14.3%)
Zone III	1/21 (4.8%)
Corneal abscess	8/21 (38.1%)
Hypopyon	15/21 (71.4%)
Traumatic cataract	19/21 (90.5%)
Vitreous opacity	20/21 (95.2%)
Retinal detachment	5/21 (23.8%)
Retinal lesions	12/21 (57.1%)
Choroid detachment	1/21 (4.8%)
Total	21/21 (100%)

*Not all patients had available data for each variable.

Among the 21 patients, five patients (23.8%) were lost to follow-up after discharge, and the average follow-up time was 12 ± 14.4 months (range: 1–53 months). At the last follow-up, six patients (37.5%) underwent silicone oil removal, and 10 patients were still maintained with silicone oil tamponade. Since most of the patients were young and unable to cooperate in visual acuity tests, only 10 patients had available visual acuity data in the last follow-up, and all the visual acuity results were no better than counting fingers.

The causative microorganisms are shown in Table 3. Among the 21 isolates, 10 (47.6%) were *S. pneumoniae*, nine (42.9%) were viridans group streptococci and two (9.5%) were *S. pluranimalium*. In the different age groups, viridans group streptococci were the most common isolates, accounting for 58.3% of cases in patients aged 0–3 years, but only 22.2% of cases in patients aged 4–6 years (*p* = 0.18). The proportion of *S. pneumoniae* was as high as 66.7% in patients aged 4–6 years, but only 33.3% in patients aged 0–3 years (*p* = 0.20).

**Table 3:** The distribution of causative microorganisms confirmed by culture.

	Patients aged 0–3 years	Patients aged 4–6 years	Number	Percentage
*Streptococcus pneumoniae*	4/33.3%	6/66.7%	10	47.6%
*viridans group streptococci*	7/58.3%	2/22.2%	9	42.9%
*Streptococcus pluranimalium*	1/8.3%	1/11.1%	2	9.5%
Total	12	9	21	100.0%

The antibiotic susceptibilities of streptococcus in this study are summarised in Table 4. All the 21 isolates were sensitive to cefuroxime (100%), and the susceptibility rate to ceftazidime and cefazolin reached 80%. The susceptibility rate of streptococcus to chloramphenicol was 100%, while that to levofloxacin and ofloxacin were 95.0% and 90.5%, respectively. The susceptibility rate of streptococcus to penicillin was 62.5%, and that of the aminoglycoside antibiotics such as neomycin, tobramycin and amikacin was as low as 30% (3/10), 19.0% (4/21) and 18.8% (3/16), respectively.

**Table 4:** Susceptibility rate of isolated bacteria to different antibiotics in paediatric endophthalmitis.

	*Streptococcus pneumoniae*	*Streptococcus viridans*	*Streptococcus pluranimalium*	Total specimen
Levofloxacin	9/10	8/8	2/2	19/20
	90.0%	100.0%	100.0%	95.0%
Ofloxacin	9/10	8/9	2/2	19/21
	90.0%	88.9%	100.0%	90.5%
Ceftazidime	1/1	2/2	1/2	4/5
	100.0%	100.0%	50.0%	80.0%
Tobramycin	1/10	1/9	2/2	4/21
	10.0%	11.1%	100.0%	19.0%
Neomycin	1/4	1/4	1/2	3/10
	25.0%	25.0%	50.0%	30.0%
Cefuroxime	10/10	9/9	2/2	21/21
	100.0%	100.0%	100.0%	100.0%
Cefazolin	1/1	2/2	1/2	4/5
	100.0%	100.0%	50.0%	80.0%
Chloramphenicol	1/1	2/2	2/2	5/5
	100.0%	100.0%	100.0%	100.0%
Penicillin	6/9	4/7	-	10/16
	66.7%	57.1%	-	62.5%
Amikacin	1/9	2/7	-	3/16
	11.1%	28.6%	-	18.8%
Cefoxitin	4/9	5/9	-	9/18
	44.4%	55.6%	-	50.0%
Azithromycin	0/3	1/2	-	1/5
	0.0%	50.0%	-	20.0%

## Discussion

In this study, we analysed the clinical data of 21 preschool children with post-traumatic endophthalmitis caused by streptococcus species in southern China. Our study showed a higher proportion of males than females, and *S. pneumoniae* was the most common isolate. However, the dominant isolates differed between the 0–3-year-old group and 4–6-year-old group. Streptococcus was sensitive to chloramphenicol, fluoroquinolone and cephalosporin. Despite aggressive treatment, the final visual acuity did not exceed counting fingers.

Previous studies on paediatric ocular trauma found that the proportion of males was close to that of females in younger children and increased with age [19–21]. Preschool patients aged 0–6 years were included in our study, and the proportion of males was close to that of females (57.1% vs. 42.9%), consistent with the findings of previous studies. Gunes et al. reported that open globe injuries in preschool children (≤6 years) occurred most frequently at home (45.4%), and the most common cause of injury was kitchen items [22]. Similarly, our study found that over 85% of injuries occurred at home were mostly self-inflicted, and that scissors were the leading cause of injuries (28.6%). Zone I was the most common wound entry site, and traumatic cataract presented as the common sign in our study, consistent with previous studies on paediatric post-traumatic endophthalmitis [1, 21].

The most common isolates were varied in streptococcal endophthalmitis studies, where most participants were adults. Yospaiboon found that *S. pneumoniae* was dominant in 45 cases, while Kuriyan reported that viridans group streptococci were dominant in 63 cases [8, 11]. Our study found that *S. pneumoniae* was more prevalent than viridans group streptococci in paediatric post-traumatic endophthalmitis (47.6% vs. 42.9%, respectively). We also found that the main isolates differed between the two age groups. Viridans group streptococci were the main isolates of post-traumatic streptococcal endophthalmitis in children aged 0–3 years, while *S. pneumoniae* was dominant in children aged 4–6 years. Viridans group streptococci are a heterogeneous group of organisms that can be human commensals, colonising the gastrointestinal and genitourinary tracts in addition to the oral mucosa [23]. Younger children, aged 0–3 years, were more likely to bite and scratch objects while playing, leading to viridans group streptococci infection through the mouth-object-eye route, which could explain the higher proportion of viridans group streptococci infection in this age group.

Vitrectomy can remove bacterial and inflammatory debris, preventing further damage. Previous studies found that vitrectomy combined with silicone oil tamponade could improve the anatomical and functional results in post-traumatic endophthalmitis in an adult-dominant population [24]. Jin et al. analysed the visual outcomes of post-traumatic endophthalmitis in patients aged 0–15 years and found that, following combined vitrectomy and silicone oil tamponade, visual acuities were not only favourable but also often better than those predicted by Ocular Trauma Scores [25]. In our study, all the patients underwent vitrectomy combined with silicone oil tamponade, and the infection was successfully controlled with an average of 1.8 ± 2.5 days from admission to surgery. However, the visual outcomes were unsatisfactory at the last follow-up. There are several reasons for this finding. First, streptococci are virulent bacteria that can cause severe inflammation. Although the viridans group streptococci are relatively lower in virulence compared with other streptococci, recent studies found that streptococcus species did not significantly influence the visual outcomes, and despite prompt treatment, most patients had poor outcomes [8, 9, 11]. Second, the patients included in our study were preschool children with an average age of 3.3 ± 1.7 years. The average duration of injury-to-primary repair was 2.6 days with 19.1% of patients presenting a self-sealed wound, and 57.1% of patients presenting typical signs of endophthalmitis at the time of primary repair. Young children may find it difficult to describe their symptoms clearly after injury, leading to delayed diagnosis and treatment of endophthalmitis, which might explain the poor visual outcomes despite prompt treatment upon admission [1, 15]. Previous studies on adult-dominated streptococcal endophthalmitis patients showed that 24.7–33.3% of cases resulted in evisceration or enucleation [8, 11]. In our study, infections were under control after vitrectomy combined with silicone oil tamponade, and there was no requirement for evisceration or enucleation during the follow-up period, even in patients who had vision with no light perception or those with slight atrophy of the eyeball.

Kuriyan et al. found that the susceptibility rates of streptococcus to ceftriaxone, vancomycin and levofloxacin were 98%, 95% and 93%, respectively, in the adult-dominated streptococcal endophthalmitis [8]. Jones et al. reported the drug sensitivity of *S. pneumoniae* in the United States and showed a sensitivity rate of 98.8% to levofloxacin and 99.1–100% (susceptibility depending on breakpoint criteria) to ceftaroline [26]. In our study, streptococcus was sensitive to cephalosporins and fluoroquinolones, such as cefuroxime and levofloxacin (100% and 95.0%, respectively), consistent with the previous studies. *S. pluranimalium* is a species of streptococcus causing human infection reported in recent years [27]. Ganesh et al. recommended vancomycin, aminoglycoside and cephalosporin as the preferred antibiotics to treat *S. pluranimalium* infections [28]. Although only two isolates of *S. pluranimalium* were identified in our study, the two isolates were susceptible to levofloxacin, ofloxacin, tobramycin and cefuroxime. Therefore, to treat paediatric post-traumatic endophthalmitis caused by streptococcus, the topical use of antibiotics such as levofloxacin and cephalosporin eye drops are recommended. Considering the restriction on the systemic use of quinolones in children, cefuroxime is recommended for intravenous antibiotic treatment.

This study focused on the clinical features, antibiotic susceptibilities and outcomes of post-traumatic endophthalmitis caused by streptococcus in preschool children. However, there are also limitations arising from the retrospective nature of this study. The data, particularly the follow-up data, were incomplete. The patients in our study came from all over China, rendering the follow-up difficult because of distance. Young children hardly cooperated with the visual acuity test, especially in the best-corrected visual acuity test. In addition, because of the small sample size, some antibiotic susceptibilities were only tested in two strains in the subgroup, increasing the likelihood of bias.

In conclusion, we analysed the clinical characteristics, drug sensitivity and outcomes of post-traumatic streptococcal endophthalmitis in preschool children. *S. pneumoniae* was the most common isolate; however, viridans group streptococci were dominant in younger children aged 0–3 years. Both cefuroxime and levofloxacin showed excellent susceptibility and are thus recommended for treatment. Although the infection was under control after prompt treatment upon diagnosis, the vision outcomes were still poor.

## Summary

### What was known before

- Streptococcus species is the leading cause of endophthalmitis after glaucoma filtering surgery, and is also the common genus of organisms identified in endophthalmitis post cataract surgery and post intravitreal injection in adult-dominated population.
- Despite prompt treatment, the visual outcomes of streptococcal endophthalmitis in adult-dominated population were mostly poor, and about 24.7–33.3% of cases resulted in evisceration or enucleation.
- Streptococcus was a common cause of post-traumatic endophthalmitis in children.

### What this study adds

- *S. pneumoniae* was the most common isolate in general; however, viridans group streptococci were dominant in younger children aged 0–3 years.
- Both cefuroxime and levofloxacin demonstrated excellent susceptibility, and are thus recommended for treatment.
- Although the infection was under control after prompt treatment upon diagnosis, the vision outcomes were still poor.
